# Hoarseness and Loss of Voice

**Published:** 1887-09

**Authors:** Barclay J. Baron

**Affiliations:** Physician in charge of the Throat Department of the Bristol General Hospital


### HOARSENESS AND LOSS OF VOICE.

BY

BARCLAY J. BARON, M.B. EDIN.,

*Physician in charge of the Throat Department of the Bristol General Hospital.*

---

IN the term "hoarseness" I include not only those coarse changes in the voice which render it rough and husky, but also those finer ones, by which it is altered in *pitch* as well as in *clearness*. Owing to these changes, a person who normally sings tenor songs is unable any longer to do so, because the highest notes first lose their clearness, and then are lost altogether, the compass being thereby lessened; along with this, it is found that all the notes that are left have lost their clearness and tone, and fatigue of voice very soon ensues, after an attempt is made to sing.

Now, although there is this most noticeable interference with the power and range of the *singing voice*, there may be nothing abnormal in the *speaking voice*, except that, perhaps, it is slightly deepened; it is not, in other words, *hoarse*, in the ordinary acceptation of that term.

It is, however, most important not to overlook this alteration of vocal power, as grave disease, quite unsuspected by the patient, and for which he therefore does not come for medical advice, may be present, and may be the cause of the change.

Hoarseness and aphonia are symptoms found in very widely different diseases, some of which affect the larynx, others affecting other parts of the body, notably the chest. These symptoms, therefore, are often of great value in throwing light on otherwise obscure cases, and if (as I believe ought to be done) a systematic laryngoscopic examination were always carried out, where these symptoms are or have been present, more accuracy of diagnosis and of treatment would be ensured.

Excluding cases of acute tonsillitis and pharyngitis, in which there was a certain amount of thickness of voice and difficulty in speaking, owing to the pain and dryness of the throat present, and of chronic pharyngitis in which there was no accompanying chronic laryngitis, I have seen over 100 cases of hoarseness or loss of voice, the majority of which have been under my care at the Bristol General Hospital.

I find from notes of these cases that they may be thus divided up in order of frequency of cause:

1. Chronic Laryngitis.
2. Hysteria.
3. Phthisis Laryngea.
4. Laryngeal Anæmia, especially in Phthisis.
5. Acute Laryngitis.
6. Laryngeal Syphilis.
7. Growths in the Larynx.
8. Aneurism.
9. Œdema of the Larynx.

My experience doubtless agrees with that of all who have had an opportunity of observing cases of throat disease, in finding that *simple chronic laryngitis* is the commonest cause of hoarseness and loss of voice. Impairment of the function of the larynx is the most striking feature of the disease, and this varies from only a slight deepening of the voice to huskiness, hoarseness, and loss of voice. Hawking and clearing the throat frequently is usually troublesome to the patient. Fatigue of voice soon supervenes after slight exertion, although it is very curious, that a person with this affection often speaks much more clearly after a few moments' use of the voice, than he did when he first began to speak.

If there is cough, it is usually hard and almost purposeless, a little pellet of mucus being expectorated after a great deal of exertion, secretion being rarely abundant.

On examining the patient with the laryngoscope, all the symptoms are explained. The whole larynx is more or less congested and swollen, the vocal cords are swollen, they vary in colour from yellow to bright red, and their free edges may be ulcerated and uneven. Mucus is seen attached to various parts of the mucous membrane, and especially to the edges of the vocal cords, this gives rise to the cough, and as it is only a small piece which sticks on the cords, it takes a good deal of coughing, before the muscles of the larynx are able to grasp it and throw it out.

The hoarseness and loss of voice are due to derangements in the mobility of the larynx, due, partly to the swollen inter-arytenoid mucous membrane, protruding between the vocal cords at their posterior ends, and so mechanically preventing their closure, partly to the fact that the ventricular bands also are swollen, and sometimes impede the true cords, added to which there is some interference with the muscular apparatus of the larynx, which is thus unable to close the glottis for purposes of speech.

The treatment consists in resting the voice as much as possible, and, as several of my cases have been those of people who use their voices a good deal, *e.g.* preachers, school teachers, singers, and street hawkers, I have found how extremely valuable this is; in fact, it is almost impossible to cure the complaint, if the excessive use of the voice goes on. Avoidance of all irritating gases, dust, peppery food, smoking, alcohol, and hot fluids, must be insisted on.

In addition to this, astringents must be locally applied to the larynx, and especially to the vocal cords; and after having tried a good many remedies, my favourite solution for brushing the vocal cords is one of chloride of zinc—grs. xv. to xxx. to ℥j. of glycerine. This application must be made every day for a week or ten days, then every other day for a week, then every third day, and so on, the voice generally becoming clear in about a month, supposing it to be an average case, and all the directions given are followed out. The astringent application ought to be continued at intervals for some time longer, and, in fact, until the vocal cords have become white or nearly so, the swelling of the laryngeal mucous membrane has subsided, and the voice is not only clear, but remains so after considerable exertion. I may add, that I believe brushing with the astringent solution that I have mentioned, is infinitely better than insufflation of alum or any other astringent powder, as it can be so much more accurately applied, and distributes itself more thoroughly.

*Hysterical Aphonia* results from paresis or paralysis of the adductors of the vocal cords, the result of this being, that on attempted phonation there is a greater or less space left between the opposed edges of the cords, through which air escapes and a whispering voice is the result.

The loss of voice usually comes on quite suddenly, and, as was the case in several of my patients, on awaking in the morning, and in many of them there was the common hysterical history of some emotional disturbance, trouble, &c., preceding it. The aphonia may be intermittent, attacking and leaving the subject of it quite suddenly. Reflex acts, such as coughing and sneezing, are normally performed, the former being often very loud, eliciting much sympathy and expressions of anxiety from injudicious friends.

Before diagnosing hysteria, the existence of deeply anæmic conditions, pulmonary phthisis, and other debilitating diseases, and thoracic tumours, ought to be excluded as far as possible. The laryngoscope must decide as to the existence of any affection of the larynx, other than the weak or paralysed condition of the muscles that close the glottis. I find that it is well to observe the vocal cords during expiration and inspiration, at rest and during phonation, by making the patient say "Ah" and "Ee;" and in a genuine hysterical case, whilst there is no such mechanical hindrance to closure of the glottis as one sees in chronic laryngitis, and whilst the vocal cords are of their usual colour, thickness, and so on, yet there is a chink of greater or less breadth left between their free edges, when speech is attempted, which shows the feebleness of the adductor muscles. The only point in the treatment worth referring to is, as to the value of endo-laryngeal Galvanism, which works wonders in some cases.

Amongst my cases of *phthisis laryngea*, there were three which were in the early stage, *i.e.* of infiltration and œdema. In one case there was swelling of the epiglottis, which was so altered in shape, as to assume the so-called "turban-like" form. This patient came to me merely complaining of soreness on swallowing; I

noticed, however, the peculiar deepening of his voice. On examining his fauces, there was nothing seen which could account for the sore throat; his lungs were distinctly diseased, and the laryngoscope revealed much œdematous swelling of the epiglottis, and also a little thickening of the mucous membrane over the arytenoid cartilages. From these appearances, and the chest condition, laryngeal phthisis could be diagnosed with confidence. I mention the case because it illustrates an axiom; viz., that *whenever there is pain on swallowing, and there are no pathological appearances of the fauces sufficient to account for the symptom, then a laryngoscopic examination ought never to be omitted.*

To allay the pain on swallowing, I have found a 10 per cent. solution of cocaine in glycerine of borax, painted on the epiglottis, is the best local treatment; along with which, all the usual measures for combating the lung mischief must of course be carried out; also, the patient ought to swallow nothing hot or irritating, nor ought he to use the voice too much.

In 12 other cases the disease had advanced to the stage of ulceration of epiglottis, laryngeal mucous membrane, and vocal cords. The symptoms varied according to the part attacked, *e.g.* hoarseness being prominent if the cords are attacked, soreness on swallowing if the epiglottis is diseased, and a peculiar deep voice if there is swelling and slight ulceration only of laryngeal mucous membrane.

In most cases of laryngeal phthisis, there is pain on swallowing, which may be so great as to induce the patient to almost starve, rather than submit to it. Here it is manifestly of first importance, if the patient is to be well nourished, that this symptom should be allayed; and many things have been tried in order to this. There is no doubt but that cocaine solutions, brushed or sprayed on to the affected parts, have driven every other pain-stopping drug out of the field, and for brushing I use them strong, viz., 10 to 20 per cent., and the result is extremely gratifying—a good meal being eaten painlessly after their use, when previously water could not be drunk without pain. Insufflation of oxychloride of bismuth and morphia, and inhalations of the compound tincture of benzoin and oil of eucalyptus, also soothe. In order to keep the ulcers clean and promote healing, I like insufflations of iodoform, and inhalations of creasote and terebene.

Lastly, there are two new remedies which I have tried; viz., 20 per cent. solution of menthol in olive oil, dropped on to the parts. From this I cannot acknowledge to have derived much help; its pain-destroying and healing properties being, as far as my experience goes, very small and very transient. The other is lactic acid, of which I have used solutions of various strengths—at first 10 per cent., and gradually increasing to 70 per cent.; and I believe that it cleans the ulcers, and promotes healing and cicatrisation. It is apt to set up a good deal of spasm of the glottis, and I have been a little alarmed, once or twice, at the semi-suffocated condition into which patients have got, owing to this; otherwise I have experienced no trouble from its use. In one case, where there were a number of polypoid excrescences on the inter-arytenoid mucous membrane, its application in strong solution used to free the patient from pain, on swallowing and talking, for two or three days; and he always asked me to apply it on that account, as it seemed to render the parts almost anæsthetic, probably owing to a slight caustic action. It also did good in this case by decreasing the size of the elevations.

Scarifying of the infiltrated parts is practised by some surgeons; but I have never seen any great advantage accrue from this, and it is certainly not devoid of the risk of setting up increased inflammation and ulceration.

Besides all these measures, of course the usual treatment of pulmonary phthisis must be gone on with.

In one case I saw necrosis of the right arytenoid cartilage come on, and was able to promote healing by clearing out the *débris* and stuffing the ulcer with iodoform; and although this patient has a small crater-like opening at the site of the formerly ulcerated ragged hole, and although the mucous membrane surrounding this opening is still swollen, there was no sign of the extension of the mischief when last I saw him, and I believe that removing the *débris* with a probe and using iodoform did great good in this case.

My cases of *acute laryngitis* showed the usual appearances on examination with the laryngoscope; viz., bright-red colour of the mucous membrane of the larynx, including the vocal cords, along with some swelling of these parts.

Two of them were in little children, in whom, owing to the small calibre of the throat, there is no small danger of suffocative attacks from mucus drying on the swollen parts. The usual treatment—viz., frequent hot poultices, steam inhalations of the compound tincture of benzoin, a very moist warm atmosphere, and warm drinks, along with salines and remedies to keep down the cough—soon cured the condition.

Besides the cases of *syphilis of the larynx*, which were associated with evident hoarseness or aphonia, I have seen others in which, along with secondary syphilis of the pharynx, there was an obstinate erythematous blush on the laryngeal mucous membrane; but this caused no evident alteration of voice, and got quite well under the usual treatment.

Several of my cases of chronic laryngitis occurred in syphilitic people; but I see no sufficient reason for believing this to be other than a mere coincidence.

In one of my patients, along with extensive destruction of the epiglottis, there were gummata of various conditions on that cartilage, and by frequent laryngoscopic examinations I was able to watch these go through the various stages of deposition, softening, and ulceration. There was terrible pain on eating in this case, and after trying insufflations of morphia, I used cocaine; and the superiority of the latter drug over the former was most evident, the man being able to eat a good dinner in comparative comfort after its use.

In another case, along with aphonia there was evident laryngeal obstruction, the breathing being characteristically noisy; the mirror revealed large outgrowths of the mucous membrane of the larynx, in various positions, one of which, of the size of a small bean, was situated between the arytenoid cartilages, and so prevented the vocal cords coming into apposition on phonation, and thus destroyed audible voice. The mobility of the vocal cords in this case was greatly impaired and they were enormously thickened; in fact, I have never seen them so thick in any other patient.

I cauterised these outgrowths with chromic acid fused on the end of a probe, but found that whilst it removed pieces of them, it set up laryngitis, and so had to be discontinued. It was surprising how much good, in clearing the voice and also, by reducing the swelling, in allowing free and noiseless breathing, was caused by the use of iodide of potassium in big doses; and I

abandoned all local treatment, which seemed to do more harm than good.

In another patient there was scarcely any epiglottis left, owing to ulceration, and yet the man rarely got any food or liquid into his trachea, so great is the power of the parts—especially, I believe, of the ventricular bands—in adapting themselves to act as guards to the entrance to the larynx.

Here little could be done, except to alleviate pain by the measures already suggested, and I only mention the case in order to illustrate the danger in which such patients live—this man having been killed by swallowing a piece of bread and butter, and washing it down into his larynx with tea, death being caused by suffocation before a medical man, who was at once summoned, could come to his assistance.

In tertiary syphilis of the larynx, I have come to the conclusion that, beyond general antisyphilitic treatment, soothing inhalations, such as the compound tincture of benzoin, to keep down cough, and insufflations of iodoform, inhalations of creasote and eucalyptus to clean ulcers, and insufflations of morphia, or, still better, brushing and spraying the larynx with solutions of cocaine to keep down pain, little can be done; the need for tracheotomy, however, must always be kept in view.

Of the cases of *growths in the larynx*, four were innocent and one malignant. Of the four innocent growths, two were sessile papillomata; in both cases the warts were seated on the free edge of the left vocal cord, were multiple and spread over about one-third of the total length of the cord.

One case was that of a schoolmistress, who had been also teaching singing, whose larynx was necessarily exposed to a great deal of work. From her history, she had evidently had chronic laryngitis for some time. From being hoarse, she became aphonic, and had been in that condition for some months before seeing me. With the laryngoscope I found a sessile irregular growth on the anterior third of the left vocal cord, her right cord, against which this growth pressed on phonation, being in a state of congestion. Seeing that the base of the growth was so large, I resolved to try local astringent applications. I began with one of chloride of zinc, grs. xv. to the ounce of glycerine, and increased the strength of this gradually up to grs. xxx. ad ℥j; very soon the aphonia gave place to hoarseness. This became less marked, and she resumed her occupation with a husky voice. I then used solutions of lactic acid, beginning with 20 per cent. and reaching 60 per cent. strength. This set up a good deal of spasm when applied with the brush; but the growth became much smaller, looked harder and less succulent, and her voice has so far returned and become clear, that she was offered a situation as head mistress of a Board school not many weeks ago.

In another similar case the chloride of zinc solution did me equally good service.

In two of my other cases the growth was distinctly pedunculated, looking like a small red currant attached to one of the vocal cords. Both were in people who use the voice a great deal—one a street hawker, the other a comic singer. The latter case is of extreme interest, as showing how chronic laryngitis leads to the formation of growths. This man consulted me at the Bristol General Hospital about eighteen months ago for hoarseness, and I found that he was suffering from chronic laryngitis. I painted his throat with astringents and so on, and he got well. He then followed his "profession," which was that of singing in public-houses, and no doubt his larynx again became inflamed, and this led, as it will do in a certain number of patients, to growth-formation, probably papillomatous.

Both these cases were admirable ones for removal ot the growths by forceps; but unfortunately both of them got alarmed at the prospect, and so in all probability have got their hoarseness and growths in their throats to this day.

The fifth case was of great interest, as it was epitheliomatous.

The patient, a man 47 years of age, came to me in March, 1886, having had pain on swallowing for five weeks, the pain shooting up to the left ear; his voice was deepened, and he had a hard lump as big as a hazel-nut below the left ear and another below the left jaw. On examination, there was some chronic pharyngitis and considerable congestion, and some swelling of the mucous membrane of the larynx on the left side. Looking to the presence of the hard enlarged glands about the jaw on the same side as the congestion of the larynx, I considered the case suspicious of malignant disease, as syphilis was denied, and the normal condition of the lungs, which I carefully examined, excluded phthisis.

The man got easier under treatment, and I did not see him again for seven months, and then he was sent to the Bristol General Hospital by Dr. Dowson, on account of great difficulty in breathing, which it was considered, and rightly so, might necessitate tracheotomy at any time.

I found the formerly congested part occupied by a ragged epitheliomatous growth; there was great increase in the size of the glands of the neck on the same side as before, and the patient was in such a condition that I took him into the hospital. Here our House-surgeon, Dr. Newnham, had to do tracheotomy not long after his admission, and eventually the man died with malignant mischief of larynx, œsophagus, pleura, and liver.

The case is one in which I considered no operation would have been of much value, seeing that before the mischief was pronounced in the larynx, and before one could diagnose it definitely, the glands were infiltrated, besides which the operation must have been extirpation of half or of the whole larynx, and so of the severest character.

*Thoracic Aneurism* caused hoarseness in three cases which I saw: in one the right innominate artery was involved, in another the innominate and aorta, and in a third the exact position of the sac was doubtful, as there was no *post-mortem* examination. In each case the recurrent laryngeal nerve corresponding to the side on which the dilated artery compressed it was paralysed, and the vocal cord moved by the muscles which were set in action by this nerve was motionless on phonation.

The treatment in such cases is that of the aneurism; and in one case the vitality of the nerve was so far restored on removing the pressure exerted by a large right innominate aneurism, that as the sac got smaller, and the pulsation in it became less, the voice improved very much. The reason of the improvement could be seen, on laryngoscopic examination, to be due to the muscles supplied by the nerve recovering their power of approximating the vocal cords, and hence fairly clear voice resulted.

I have had two cases of *œdema of the larynx* under my care at the Bristol General Hospital: one was extraordinarily acute, the voice being completely lost in an hour or two from the beginning of the attack. Only the epiglottis, which was enormously swollen, was affected. Under very hot poultices frequently renewed, hot steam constantly inhaled, impregnated with the compound tincture of benzoin, and careful avoidance of all draughts of air, and feeding on warm milk and beef-tea in small quantities and often, I saw the epiglottis get thinner and thinner day by day, and more and more of the glottis came gradually into view—the patient completely recovering. I hope to publish the case *in extenso*, as it shows that such patients get quite well without running the risk incurred by scarification of the swollen œdematous part, which would in my patient have been very dangerous indeed.

The other case, also under my care at the Bristol General Hospital, was one of œdema of the glottis, and also of parts below the glottis. Knowing that these cases of sub-glottic œdema are very often syphilitic, I put her on iodide of potassium, and on several occasions leeches were applied over the thyroid cartilage, besides which hot poultices, steam inhalations, and careful feeding were persevered with, and she got perfectly well, both as regards her voice, which when first I saw her was nearly gone, and also as regards the condition of her larynx as seen with the mirror.

Lastly, I wish to say how convinced I am of the importance of examining with the laryngoscope every case of pulmonary phthisis in which there is deepening of the voice, hoarseness, or aphonia, as I have found that in many of the cases the symptom depends merely on *anæmia*; and the knowledge that this is so, has a bearing on the prognosis to which I need scarcely refer.

